# A PAS domain-containing regulator controls flagella-flagella interactions in *Campylobacter jejuni*

**DOI:** 10.3389/fmicb.2015.00770

**Published:** 2015-07-30

**Authors:** Mark Reuter, Paula M. Periago, Francis Mulholland, Helen L. Brown, Arnoud H. M. van Vliet

**Affiliations:** ^1^Institute of Food Research, Gut Health and Food Safety ProgrammeNorwich, UK; ^2^Departamento Ingeniería de Alimentos y del Equipamiento Agrícola, Campus de Excelencia Internacional Regional “Campus Mare Nostrum,” Escuela Técnica Superior de Ingeniería Agronómica, Universidad Politécnica de CartagenaCartagena, Spain; ^3^Instituto de Biotecnología Vegetal, Campus de Excelencia Internacional Regional “Campus Mare Nostrum,” Universidad Politécnica de CartagenaCartagena, Spain; ^4^Cardiff School of Health Sciences, Cardiff Metropolitan UniversityCardiff, UK

**Keywords:** Campylobacter, flagella, transcriptional repression, PAS domains

## Abstract

The bipolar flagella of the foodborne bacterial pathogen *Campylobacter jejuni* confer motility, which is essential for virulence. The flagella of *C. jejuni* are post-translationally modified, but how this process is controlled is not well understood. In this work, we have identified a novel PAS-domain containing regulatory system, which modulates flagella-flagella interactions in *C. jejuni*. Inactivation of the *cj1387c* gene, encoding a YheO-like PAS6 domain linked to a helix-turn-helix domain, resulted in the generation of a tightly associated “cell-train” morphotype, where up to four cells were connected by their flagella. The morphotype was fully motile, resistant to vortexing, accompanied by increased autoagglutination, and was not observed in aflagellated cells. The Δ*cj1387c* mutant displayed increased expression of the adjacent Cj1388 protein, which comprises of a single endoribonuclease L-PSP domain. Comparative genomics showed that *cj1387c* (*yheO*) orthologs in bacterial genomes are commonly linked to an adjacent *cj1388* ortholog, with some bacteria, including *C. jejuni*, containing another *cj1388*-like gene (*cj0327*). Inactivation of the *cj1388* and *cj0327* genes resulted in decreased autoagglutination in Tween-20-supplemented media. The Δ*cj1388* and Δ*cj0327* mutants were also attenuated in a *Galleria* larvae-based infection model. Finally, substituting the sole cysteine in Cj1388 for serine prevented Cj1388 dimerization in non-reducing conditions, and resulted in decreased autoagglutination in the presence of Tween-20. We hypothesize that Cj1388 and Cj0327 modulate post-translational modification of the flagella through yet unidentified mechanisms, and propose naming Cj1387 the Campylobacter Flagella Interaction Regulator CfiR, and the Cj1388 and Cj0327 protein as CfiP and CfiQ, respectively.

## Introduction

The thermophilic *Campylobacter* species *C. jejuni* and *C. coli* are causative agents of human gastroenteritis, and are commonly transmitted via contaminated food, especially poultry meat. Diarrhoeal disease linked with *Campylobacter* spp. is prevalent in many countries in the Western world, with around half a million cases annually in the UK (Tam et al., 2012). Despite its importance as a human pathogen, much remains to be learned about *Campylobacter* virulence mechanisms. The role of the flagellar system in *Campylobacter* virulence cannot be understated. Firstly, flagellar motility confers the ability to swim toward intestinal epithelial cells, which is critical for subsequent cell invasion (Lee et al., 1986; Szymanski et al., 1995). Secondly, the flagellar Type III secretion system is utilized to secrete non-flagellar effector proteins, which have roles in virulence (Konkel et al., 2004; Song et al., 2004; Poly et al., 2007; Barrero-Tobon and Hendrixson, 2012, 2014; Neal-McKinney and Konkel, 2012). Thirdly, motility is essential for chemotaxis, and various chemotaxis-defective mutants are attenuated in animal models of disease (Takata et al., 1992; Yao et al., 1997) or show reduced immunopathology (Bereswill et al., 2011). Flagella are also required for autoagglutination [i.e., aggregation, AAG (Golden and Acheson, 2002)] in *Campylobacter*, which is thought to play a role in adherence to, and invasion of human embryonic intestinal cells (Misawa and Blaser, 2000). AAG activity is also associated with virulence in *Escherichia coli, Helicobacter pylori*, and *Burkholderia pseudomallei* (Cole et al., 2004; Boddey et al., 2006; Moreira et al., 2006).

The *Campylobacter* flagellum consists of two highly similar flagellin sub-units, FlaA, and FlaB (Guerry et al., 1991), and is heavily glycosylated, which plays an important role in flagella structure and function in *Campylobacter*. The flagellum is modified by covalent O-linked attachment of modified pseudaminic or legionaminic acid sugars (Thibault et al., 2001; Logan et al., 2002; McNally et al., 2007) and glycosylation has been shown to be essential for flagella assembly (Goon et al., 2003; Asakura et al., 2013). Moreover, subtle changes in glycosylation affect AAG as mutants unable to modify flagella with an acetamidino-derivative of pseudaminic acid, fail to autoagglutinate, suggesting that these sugar modifications are required for flagella interaction during AAG (Guerry et al., 2006). Genes encoding the biosynthetic pathway for glycan synthesis and transfer are highly variable among *Campylobacter* strains (Guerry et al., 2006), which likely gives rise to a high level of glycan heterogeneity within the genus that may have a role in antigenic variation and immune avoidance.

The whole flagellar apparatus involves the coordinated assembly of 40–100 proteins (Chen et al., 2011; Lertsethtakarn et al., 2011), and flagellar rotation imposes an energy demand on cells. Accordingly, expression of flagella genes is tightly regulated, with genes involved in the secretion apparatus subject to expression from σ^54^-dependent promoters, and the major flagellin and several effectors requiring σ^28^ (Nuijten et al., 1990; Guerry et al., 1991; Carrillo et al., 2004; Wosten et al., 2010). Other factors involved in regulating transcription of flagellar genes are the two-component FlgSR system (Hendrixson and DiRita, 2003; Wosten et al., 2004) and the FlgM anti-sigma factor (Wosten et al., 2010). Flagella may be further regulated via phase variation as a result of polymeric A/T tracts within the *flgR* gene (Hendrixson, 2006). Activation of the FlgS histidine kinase is thought to be dependent on interaction with components of the flagellar assembly apparatus (Boll and Hendrixson, 2013) and σ^54^-dependent flagella genes are activated by low pH (Le et al., 2012). Glycosylation may also be regulated at the metabolic level as pyridoxal-5′-phosphate production results in decreased flagellar glycosylation (Asakura et al., 2013).

The *Campylobacter* genome encodes other environmental sensing modules, including those involved in chemotactic sensing (Marchant et al., 2002) and gene regulation (Raphael et al., 2005). Among the repertoire of sensing modules, PAS domains have been linked to both chemotaxis sensors (Reuter and van Vliet, 2013) and two-component sensors (Luethy et al., 2015). PAS (Per, Arnt, Sim) domains are wide-spread in bacteria, archea, and eukaryotes, and have roles in sensing a wide range of stimuli including light, oxygen, redox potential, and even play a role in circadian regulation in higher eukaryotes (Taylor and Zhulin, 1999). In *C. jejuni*, three PAS-domain-containing proteins are involved in balancing energy and redox taxis (Elliott and Dirita, 2008; Reuter and van Vliet, 2013). Here we investigate the function of the previously uncharacterized PAS-domain protein Cj1387c in *C. jejuni*. Using a combination of mutational analysis, microscopy and proteomics, we have identified two proteins that have a role in mediating cell-cell contacts via flagella. The role of these proteins in virulence is also assessed using the *Galleria mellonella* invertebrate infection model.

## Materials and methods

### *C. jejuni* strains and growth conditions

*Campylobacter jejuni* strain NCTC 11168 and its isogenic mutants (Table 1) were routinely cultured in a MACS-MG-1000 controlled atmosphere cabinet (Don Whitley Scientific) under microaerobic conditions (85% N_2_, 5% O_2_, 10% CO_2_) at 37°C. For growth on plates, strains were either grown on Brucella agar, blood plates [Blood Agar Base 2 (BAB), 1% yeast extract, 5% horse blood (Oxoid)], or BAB with Skirrow supplements (10 μg ml^−1^ vancomycin, 5 μg ml^−1^ trimethoprim, 2.5 IU polymyxin-B). Broth culture was carried out in Brucella broth (Becton Dickinson).

**Table 1:** **Bacterial strains described in this study**.

**Strain**	**Description^a^**
***E. coli* STRAINS**	
Top10	General cloning strain
BL21 (DE3)	Over-expression strain compatible with the pET expression system (T7 promoter)
BL21 (DE3)::pCASO40	Cj1388 expressed in pET28a resulting in an N-terminal his-tagged protein
BL21 (DE3)::pCASO53	Cj1388^Cys71Ser^ expressed in pET28a resulting in an N-terminal his-tagged protein
***C. jejuni* STRAINS**	
NCTC 11168	Wild-type *C. jejuni* (Parkhill et al., 2000)
11168 Δ*cj1387c*	NCTC 11168 *cj1387c::cat*^*R*^
11168 Δ*cj1387cKm*	NCTC 11168 *cj1387c::kan*^*R*^
11168 Δ*cj1388*	NCTC 11168 *cj1388::kan*^*R*^
11168 Δ*cj0327*	NCTC 11168 *cj0327::cat*^*R*^
11168 Δ*cj1388cj0327*	NCTC 11168 *cj1388::kan*^*R*^ *cj0327::cat*^*R*^
11168 Δ*flaAB*	NCTC 11168 (*cj1338-39c*)*::kan*^*R*^ (Reuter and van Vliet, 2013)
11168 Δ*flaABcj1387c*	NCTC 11168 *flaAB::kan*^*R*^ *cj1387c::cat*^*R*^
11168 Δ*pflA*	NCTC 11168 *cj1565c::kan*^*R*^
11168 Δ*pflAcj1387c*	NCTC 11168 *cj1565c::kan*^*R*^ *cj1387c::cat*^*R*^
11168 Δ*cj1387c*::*cj1387c*^fdxApr^^*^	NCTC 11168 *cj1387c::cat*^*R*^ *cj0046*::*cj1387c*^fdxApr^^*^kan^*R*^
11168 Δ*cj1387c*::*cj1388*^fdxApr^^*^	NCTC 11168 *cj1387c::cat*^*R*^ *cj0046*::*cj1388*^fdxApr^^*^kan^*R*^
11168 Δ*cj0327*::*cj0327*^fdxApr^^*^	NCTC 11168 *cj0327::cat*^*R*^ *cj0046*::*cj0327*^fdxApr^^*^kan^*R*^
11168*::cj1387c*^fdxApr^^*^	NCTC 11168 *cj0046*::*cj1387c*^fdxApr^^*^kan^*R*^
11168 Δ*cj1388*::cj1388^*^	NCTC 11168 *cj1388::kan*^*R*^ *cj0046*::cj1388^*^cat^*R*^
11168 Δ*cj1388*::*cj1388*^*cys*71ser^^*^	NCTC 11168 *cj1388::kan*^*R*^ *cj0046*::*cj1388*^*cys*71ser^^*^cat^*R*^

a kan^R^, kanamycin antibiotic resistance cassette; cat^R^, chloramphenicol antibiotic resistance cassette;

* * denotes complementation construct: ^fdxApr^, gene under control of the constitutive fdxA promoter*.

### Construction of Δ*cj1387c*, Δ*cj1388*, Δ*cj0327*, Δ*cj1388cj0327*, and Δ*cj1387cflaAB* mutants

Plasmids used in this study are listed in Table 2, and primers used are listed in Supplementary Table 1. DNA fragments of the target gene and approximately 500 bp of flanking sequence on each side were PCR amplified using Phusion DNA polymerase (New England Biolabs) using the primers detailed in Supplementary Table 1. These amplified fragments were purified (PCR purification kit, QIAGEN, Germany), digested and ligated into pNEB193 (New England Biolabs) also digested with the corresponding restriction endonucleases (see Supplementary Table 1) and transformed into a chemically competent *E. coli* strain Top10 (Invitrogen). Constructs containing an insert were selected on LB agar plates containing 100 μg ml^−1^ carbenicillin and 20 μg ml^−1^ X-gal. Constructs containing an insert were confirmed by restriction digest analysis and sequencing (Eurofins MWG Operon, Ebersberg, Germany). To make the Δ*cj1388* and Δ*cj0327* insertional inactivation constructs, *Bam*HI sites, were introduced at the 5′ and 3′ end of the target genes by inverse PCR using primers detailed in Supplementary Table 1. Inverse PCR products were purified (PCR-purification kit, QIAGEN) digested with *Bam*HI, and ligated to either the kanamycin cassette (*Bam*HI cohesive ends) from pMARKan9 (Δ*cj1388*) or chloramphenicol cassette (*Bam*HI cohesive ends) from pAV35 (Δ*cj0327*). All ligation reactions were transformed into *E. coli* strain Top10 and positive transformants were selected for by plating on LB agar supplemented with either 30 μg ml^−1^ kanamycin or 30 μg ml^−1^ chloramphenicol as appropriate. Plasmids with insert, and insert orientation, were verified with restriction digest analysis and sequencing. Single *C. jejuni* mutant strains were isolated after transformation of the *C. jejuni* NCTC 11168 wild-type strain with plasmids by electroporation (Reuter and van Vliet, 2013), followed by selection on plates supplemented with either 50 μg ml^−1^ kanamycin or 10 μg ml^−1^ chloramphenicol. The Δ*cj1387c* mutant (antibiotic cassette in the same orientation as *cj1387c*) was made by transforming wild-type cells with either pCj1387::KanC1 (Kan^r^) or pCj1387::CatC1 (Cat^r^). To make the Δ*cj1388cj0327* double mutant, the Δ*cj1388* strain was transformed with the *cj0327* insertional inactivation construct and transformants selected on Brucella plates supplemented with 10 μg ml-1 chloramphenicol. To make the 11168 Δ*cj1387c-flaAB* strain (*cj1387c* inactivated in a non-motile background), the Δ*flaAB* strain (Kan^r^) was transformed with pCj1387::CatC1 and transformants selected on Brucella plates supplemented with 10 μg ml^−1^ chloramphenicol.

**Table 2:** **Plasmids described in this study**.

**Plasmids**	**Description^a^**
pNEB193	General cloning vector (NEB)
pET28a	Over-expression strain for making N-terminally his-tagged proteins
pCfdxA	Complementation plasmid containing *cj0046* flanks, Chloramphenicol resistance cassette and *fdxA* promoter (Reuter and van Vliet, 2013)
pC46	Complementation plasmid containing *cj0046* flanks, Chloramphenicol resistance cassette (for native promoter complementation (Reuter and van Vliet, 2013)
pCj1387::KanC1	*cj1387c* disruption plasmid, kan^*R*^
pCj1387::CatC1	*cj1387c* disruption plasmid, cat^*R*^
pCASO24	*cj1387c* complementation plasmid—*fdxA* promoter, kan^*R*^
pCASO29	*cj1388* disruption plasmid, kan^*R*^
pCASO36	*cj0327* disruption plasmid, cat^*R*^
pUCΔpflA	*cj1565c* (*pflA*) disruption plasmid, kan^*R*^
pCASO40	*cj1388* cloned into pET28a
pCASO41	*cj1388* complementation plasmid—*fdxA* promoter, cat^*R*^
pCASO52	*cj1388*^cys71ser^ complementation plasmid—*fdxA* promoter, cat^*R*^
pCASO53	*cj1388*^cys71ser^ cloned into pET28a
pCASO62	*cj0327* complementation plasmid—*fdxA* promoter, kan^*R*^
pMARKan9	Source of Kanamycin resistance cassette (van Vliet et al., 1998)
pAV35	Source of Chloramphenicol resistance cassette (van Vliet et al., 1998)

a *kan^R^, kanamycin resistance; cat^R^, chloramphenicol resistance*.

To confirm the position of the antibiotic cassette in antibiotic resistant clones, colonies were picked into 10 ml Brucella broth, supplemented with the appropriate antibiotic, and grown overnight. Genomic DNA was isolated from 4 ml overnight culture (DNeasy kit, QIAGEN). Diluted genomic DNA was used as template for PCR using primers that anneal outside of the cloned flanking regions in combination with antibiotic cassette-specific primers (Supplementary Table 1).

### Construction of *cj1387c, cj0327*, and *cj1388* complementation constructs

*C. jejuni* mutants were complemented by inserting the *cj1387c, cj0327*, or *cj1388* gene *in trans* using the *cj0046* pseudogene (Reuter and van Vliet, 2013). The genes were PCR-amplified from the NCTC 11168 genomic DNA (for primers see Supplementary Table 1), digested with *Nco*I or *Bsp*HI, and ligated into pKfdxA or pCfdxA digested with *Esp*3I (Fermentas) to make pCASO24 (*cj1387c* expressed from constitutive *fdxA* promoter), pCASO62 (*cj0327* expressed from constitutive *fdxA* promoter), and pCASO41 (*cj1388* expressed from constitutive *fdxA* promoter). These constructs were transformed into the relevant mutant backgrounds using standard electroporation methods. Complementation strains were confirmed by PCR using purified genomic DNA and primers that anneal outside of the *cj0046* flanking regions in combination with gene-specific primers (see Supplementary Table 1). To make the cys70ser substitution, pCASO41 was used as template for inverse PCR using primers 1388Cys-SerDpnI1/2 (see Supplementary Table 1). The PCR product was digested with *Dpn*I for 60 min at 37°C and then purified (PCR purification kit, QIAGEN). A 1/10 volume of the total purified DNA was transformed into *E. coli*. To confirm those constructs with the correct sequence, plasmid DNA was purified and sequenced using the T7 primer (Eurofins Genomics, Ebersberg, Germany).

### Assessment of growth

A 50 μl single-use glycerol stock, routinely stored at −80°C, was used to inoculate a BAB plate with Skirrow supplements and these cells were used to inoculate fresh Brucella broth. Cultures were grown in microaerobic conditions with shaking overnight at 37°C. The overnight culture was diluted to A_600_ ≈0.05 (~ 1 × 10^7^ CFU ml^−1^) in either 50 ml fresh Brucella broth in a T75 filter screw cap tissue culture flask (TPP, Helena Biosciences) or 200 μl in a 96-well plate [flat bottom, non-treated, sterile, polystyrene, (Corning, NY, USA)]. Flasks were grown in microaerobic conditions with shaking at 37°C and the A_600_ was monitored every 60 min for up to 10 h. Growth in 96-well plates was assessed using an Omega plate reader [FLUOstar Omega (BMG Labtech, Germany)] linked to an atmospheric control unit in microaerobic conditions at 37°C. Omega assays were run for 24 h and A_600_ data was recorded every 60 min.

### Autoagglutination assays

Autoagglutination (AAG or cell aggregation and sedimentation) was measured by monitoring the A_600_ of a 1 ml overnight culture in a plastic cuvette, statically incubated at room temperature. All strains were assessed using at least three independent biological replicates. The percentage autoagglutination (% AAG) was calculated as the recorded A_600_ divided by the initial A_600_. Data was analyzed for statistical significance using unpaired *t*-tests (GraphPad Prism 6.01).

### Light microcopy and flagella staining using the Ryu stain

Typically, 10 μl of an overnight culture was added to a microscope slide and covered with a coverslip. Cells were monitored using an Eclipse 50i microscope using the x100 lens (x1000 including ocular lens) to confirm either a motile or non-motile (Δ*flaAB*, Δ*cj1387c-flaAB*) swimming phenotypes. When necessary, videos (15 frames second^−1^, 320 × 240 pixels) were captured using a Coolpix 4500 digital camera (Nikon). Video compilations were made by extracting appropriate frames using ImageJ (Rasband, W.S., ImageJ, U. S. National Institutes of Health, Bethesda, Maryland, USA, http://imagej.nih.gov/ij/, 1997–2014) and combined using iMovie version 8.0.6.

To visualize flagella, cells were stained using the Ryu stain (Heimbrook et al., 1989). Ryu stain was prepared freshly on a weekly basis (as necessary) by mixing the two components and centrifuging (15,000 × g, 3 min, room temperature). Five ul of the stain was pipetted at the edge of the coverslip to draw the stain into the sample by capillarity. Ryu stained regions were photographed (2272 × 1704 pixels, 300 dpi) using a Coolpix 4500 digital camera. ImageJ was used to prepared montage images and apply a scale bar.

### Swarm plate assays

All swarm plate assays were carried out using square 10 mm^2^ petri plates (Sterilin) inoculated with wild-type and three test strains. Swarming in rich media was assessed using Brucella broth supplemented with 0.01% TTC and 0.4% agar (Oxoid). Plates were inoculated with 5 μl of overnight culture, and incubated in microaerobic conditions at 37°C. Swarm plates were photographed using a CCD camera and gel documentation system (U:Genius, Syngene) after 24 and 48 h and halo area measured using ImageJ. For each plate, halo size was expressed as a percentage of the corresponding wild-type and each strain was tested for significance using a one-sample *t*-test, compared to a hypothetical value of 100 (GraphPad Prism 6.01).

### *Galleria* assays

The *Galleria mellonella* infection model (Champion et al., 2010) was used to assess virulence. *G. mellonella* larvae were obtained from Livefoods.co.uk (United Kingdom). *C. jejuni* strains were resuspended from Skirrow plates, centrifuged (15,000 × g, 3 min, room temperature), resuspended in PBS and adjusted to an A_600_ = 1.0. An aliquot of wild-type cells was heated at 70°C for 5 min using a MultiGene thermal cycler (Labnet International) to provide a heat-killed control. For each test strain, 10 larvae were inoculated in the right foremost pro-leg by microinjection (Hamilton, Switzerland) with a 10 μl cell suspension (~1 × 10^6^ cells). Control groups, each containing 10 larvae, were prepared in each assay: mock injection, PBS, and heat-killed bacteria. The larvae were incubated at 37°C for 24 h and then scored for survival. Larvae that showed high levels of melanization, were unable to self-right, and showed an absence of mobility were scored as non-survivors, and percentage survival was calculated. Each strain was assessed using at least four independent experiments.

### Construction of Cj1388 over-expression constructs

The *cj1388* gene was PCR amplified from the NCTC 11168 genomic DNA using primers cj1388pET_Fwd/_Rev (see Supplementary Table 1). The PCR product was digested with *Nde*I and *Bam*HI and ligated into pET28a digested with *Nde*I and *Bam*HI to make pCASO40. To make the cys70ser substitution, pCASO40 was used as template for inverse PCR using primers 1388Cys-SerDpnI1/2. The PCR product was digested with *Dpn*I for 60 min at 37°C and then purified. A 1/10 volume of the total purified DNA was transformed into *E. coli*. To confirm those constructs with the correct sequence, plasmid DNA was purified and sequenced using the T7 primers.

### Protein expression

Expression plasmids were transformed into *E. coli* BL21 (DE3) cells (NEB). For induced protein expression, 50 ml of LB supplemented with 30 μg ml^−1^ kanamycin was inoculated with 50 μl overnight broth culture and grown at 37°C with 200 rpm shaking. The A_600_ of the cultures was monitored; when the culture reached an A_600_ of ~0.5, IPTG was added to a final concentration of 0.4 mM. Cultures were grown with shaking for a further 3 h (37°C, 200 rpm). Un-induced, and hourly induction samples were collected to monitor protein expression. Cell were harvested by centrifugation (3200 × g, 15 min, 4°C) and lysed by sonication (Soniprep 150 MSE, Sanyo). His-tagged proteins were purified using Ni-NTA Spin Columns (QIAGEN). Columns were equilibrated with NPI-10 (50 mM NaH_2_PO_4_, 300 mM NaCl, 10 mM imidazole), and washed with NPI-20 (NPI + 20 mM imidazole). Protein was eluted in NPI-500 (NPI + 500 mM imidazole). For SDS-PAGE analysis, samples were mixed with 4x LDS buffer (Invitrogen, Life Technologies) with or without β-mercaptoethanol (± reducing conditions) and resolved on 4–20% precast 10 × 10 cm gels (Expedeon, UK) in 1 × MOPS at 120 V. To visualize proteins, gels were rinsed with water and stained in InstantBlue (Expedeon) overnight. Gels were imaged using a GS800 Calibrated Densitometer (BioRad) at 63.5 μm resolution.

### Two-dimensional protein gel electrophoresis

Two-dimensional protein gel electrophoresis was conducted as described previously (Shaw et al., 2012). *C. jejuni* cells were harvested from broth culture (50 ml) by centrifugation at 3220 × *g* for 15 min at room temperature. Cell pellets were resuspended in 500 μl lysis buffer (50 mM Tris (pH 7.5), 0.3% sodium dodecyl sulfate (SDS), 0.2 M dithiothreitol, 3.3 mM MgCl_2_, 16.7 μg of RNase ml^−1^, and 1.67 U of DNase ml^−1^) and lysed (Soniprep 150 MSE, Sanyo) on ice until clear. The samples were then centrifuged (20,000 × *g*, 20 min, 4°C) to remove any unlysed cells. Total cell protein was quantified using a 2D Quant kit (GE Healthcare, UK) as per the manufacturer's instructions.

### Protein identification

Protein spots of interest were picked from 2D gels using the ProPick (Genomic Solution) and subject to in-gel trypsin digestion and LC-MS-MS as previously described (Shaw et al., 2012) except using the Proteome Discoverer program (Thermo) to convert the RAW file to Mascot Generic format for Mascot Analysis (Matrix Science).

For the protein overexpression SDS-PAGE gels, the protein bands were excised and subject to in-gel digestion as previously described (Ash et al., 2014) modified for an overnight Chymotrypsin digestion under both non-reducing, and reducing and alkylating conditions. The resulting peptides were then analyzed by LC-MS-MS on an Orbitrap Fusion (Thermo). MS Convert (Chambers et al., 2012) was used to convert the RAW file to Mascot Generic format for Mascot Analysis prior to either Mascot analysis for protein identification or Mass Matrix analysis (http://www.massmatrix.net/mm-cgi/home.py) for identification of potential disulfide bonds in the non-reduced protein bands.

### Bioinformatics analysis

Pfam [http://pfam.sanger.ac.uk/ (Finn et al., 2014)] and InterProScan [http://www.ebi.ac.uk/Tools/pfa/iprscan/ (Jones et al., 2014)] were used to search for protein domains. Domain architecture information was retrieved from Pfam. Protein sequences from either complete genome sequences or whole genome shotgun assemblies were downloaded from the Patric database (http://patricbrc.org/portal/portal/patric/Home) (Wattam et al., 2014) and a BLAST database was made using BioEdit (http://www.mbio.ncsu.edu/bioedit/bioedit.html). All BLAST searches were conducted within BioEdit. EMBL String (http://string-db.org/) (Franceschini et al., 2013) was used to find homologs of Cj1387c and investigate *cj1387c*-*cj1388* genetic structure. The RNA-seq data of the *C. jejuni* NCTC 11168 wild-type have been deposited in the Gene Expression Omnibus (GEO) and Short Read Archive (SRA) databases, and are available via GEO accession number GSE49687. Transcript levels of individual genes were expressed as Reads Per Kilobase per Million mapped reads (RPKM) values, calculated after mapping of reads using CLC Genomics Workbench v5 (CLC Bio).

## Results

### *C. jejuni* contains a PAS6 domain-containing protein that exhibits limited architectural variability

**Figure 1:**
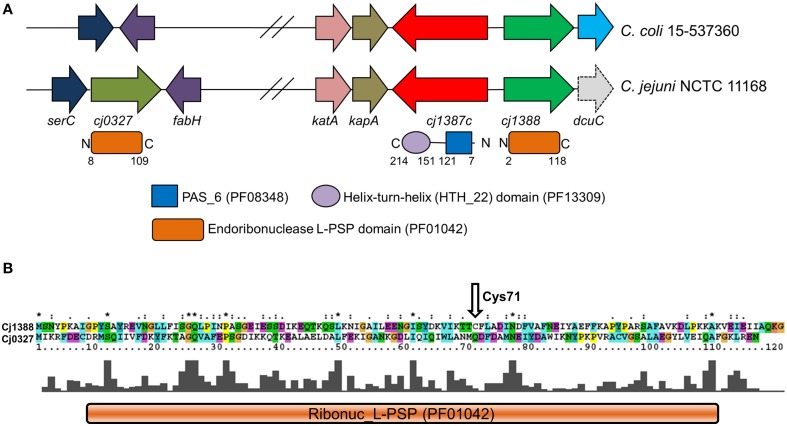
**Locus and primary sequence analysis of the** ***cj1387c*****-*****cj1388*****-*****cj0327*** **genes and encoded products**. **(A)** Diagram showing the genetic organization of the *cj0327* and *cj1387c-cj1388* genes in the *C. jejuni* and *C. coli* genomes. The gene *serC* encodes phosphoserine aminotransferase, *fabH* encodes 3-oxoacyl-[acyl-carrier-protein] synthase, and *dcuC* encodes a C4-dicarboxylate transporter protein (*dcuC* in *C. jejuni* NCTC 11168 is a pseudogene, indicated by dashed lines). **(B)** Sequence alignment of Cj1388 and Cj0327. The position of the unique cysteine residue in Cj1388 is shown. “^*^” indicates positions which have a single, fully conserved residue. “:” indicates conservation amongst similar groups of amino acids. A full explanation of the ClustalX output can be found at http://www.clustal.org/download/clustalx_help.html.

PAS domains are wide-spread signal-sensing domains involved in sensing a variety of signals including light, redox potential, oxygen, and small ligands (Taylor and Zhulin, 1999; Moglich et al., 2009). Searches of the *C. jejuni* NCTC 11168 genome sequence allowed the identification of five proteins containing predicted PAS domains. Of these, the CetB and CetC proteins, consisting of single PAS domains, and CetZ, consisting of two tandem PAS domains linked to a methyl-accepting chemotaxis domain are involved in energy and redox taxis (Hendrixson et al., 2001; Reuter and van Vliet, 2013). The Cj1491-92c two-component system has a PAS domain-containing histidine kinase, and controls expression of a gluconate dehydrogenase, and other proteins involved in heme or iron acquisition, and respiration (Luethy et al., 2015). Based on the Pfam classification of domains, all these PAS domains fall into the PAS9 family. These particular domains are found linked to a wide variety of output domains and of all the PAS families, exhibit the greatest number of architectures (Supplementary Figure 1). Further analysis of the *C. jejuni* NCTC 11168 genome revealed a protein that contained a PAS6 (YheO-like) domain linked to a helix-turn-helix domain, Cj1387c (Figure 1A). In contrast to the PAS domains found in the other *C. jejuni* signaling proteins, this member of the PAS clan is only found in prokaryotes and is represented by only five different architectures (Supplementary Figure 1) with the PAS6-Helix-turn-Helix configuration being the most abundant configuration (1816 sequences). Thus, this domain may have a specialized role distinct from signal sensing.

### Inactivation of *cj1387c* results in a flagella-mediated, tightly connected “cell-train” morphotype, which does not disrupt motility

**Figure 2:**
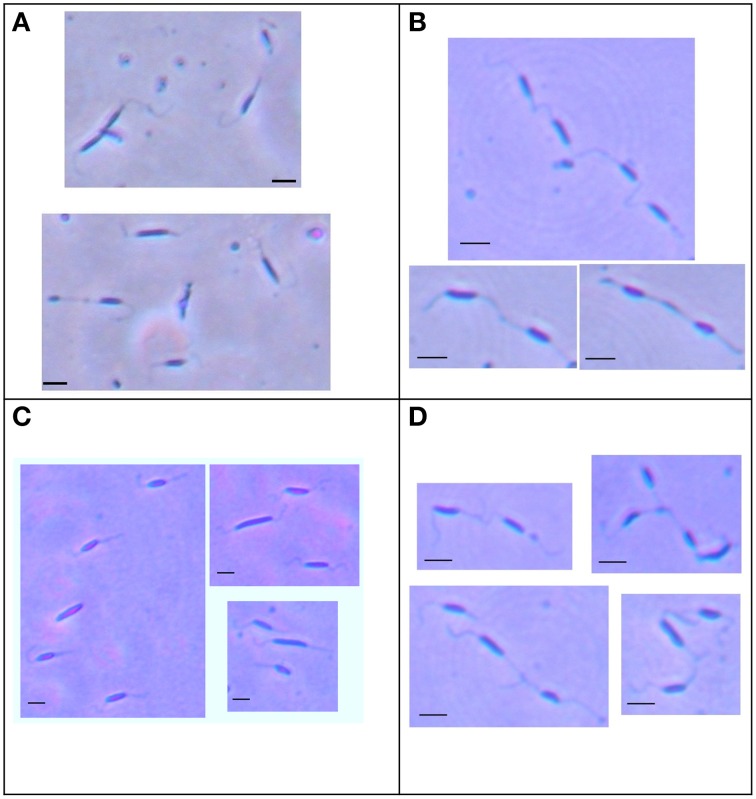
**The** ***cj1387c*** **strain forms cell chains when grown in liquid broth**. The wild-type **(A)**, Δ*cj1387c* strain **(B)**, and *cj1387c*::cj1387^*^ strain **(C)** were grown overnight in Brucella broth under microaerobic conditions at 37°C, and mounted directly on a twin-frost microscope slide. The Δ*cj1387c* strain was also subject to vortexing for 2 min before mounting **(D)**. Fresh Ryu stain was applied to the coverslip and cells photographed at × 100 magnification. Scale bar = 2 microns.

To investigate the role of Cj1387c in *C. jejuni*, the *cj1387c* gene was inactivated by insertion of an antibiotic resistance gene. The resulting strain was viable, suggesting that the Cj1387c protein does not encode an essential function, and had a wild-type growth phenotype (data not shown). When examined under light microscopy using the Ryu stain to visualize flagella, the Δ*cj1387c* cells were observed to form cell chains (“trains”) of up to four cells (Figure 2), with cells linked by their flagella. These cell chains could not be disrupted by vortexing for 2 min (Figure 2D) or extrusion through a 21 G 0.8 mm needle.

**Figure 3:**
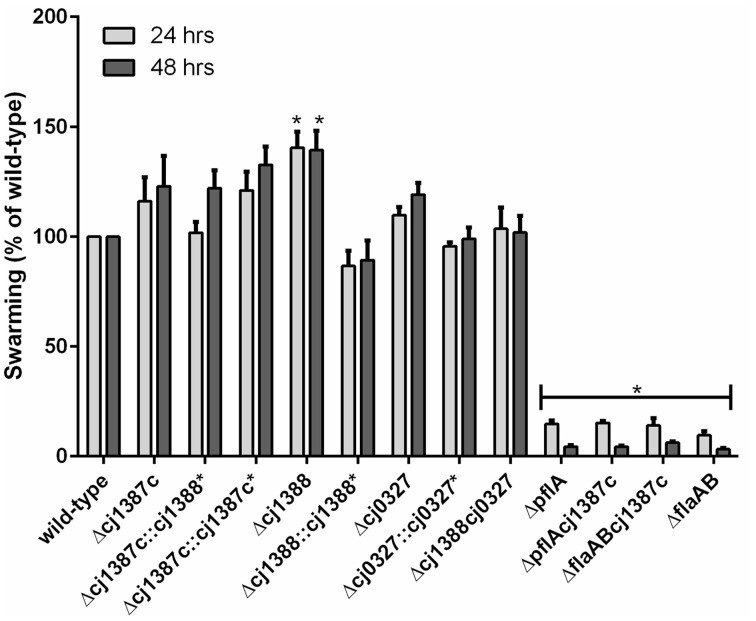
**Inactivation of the** ***cj1387c**, **cj1388***, **and** ***cj0327*** **genes does not decrease swarming motility**. Brucella broth supplemented with 0.4% agar and 0.01% TTC were inoculated with 5 μl of overnight culture and incubated in microaerobic conditions at 37°C. Halo formation was measured after 24 and 48 h and expressed as a percentage of the wild-type. Results are the mean from at least three biological replicates. An asterisk denotes statistically significant results, based on a one-sample *t*-test.

The role of flagella and the effect of the cell-train morphotype on motility were investigated by inactivation of *cj1387c* in an aflagellate Δ*flaAB* strain (Reuter and van Vliet, 2013), and the effect of flagellar rotation was investigated by inactivation of the *pflA* gene (*cj1565c*) in the Δ*cj1387c* background, which is known to result in non-functional “paralysed” flagella (Yao et al., 1994). The Δ*cj1387c* mutant displayed no defects in swimming motility when using light microscopy, and cell “trains,” containing two or three cells, were also motile (see Supplementary Video 1). Interestingly, these cell “trains” appeared to contain a dominant cell, leading the swimming cells, as gross directional changes were not observed. Swarming motility, as demonstrated using agar swarm plates, was also not significantly different from the wild-type (Figure 3). In contrast, the Δ*cj1387c-flaAB* double mutant was not motile, and halo size was less than 10% of the wild-type halo on swarm plates. The Δ*cj1387c-flaAB* cells showed no flagella or cell-cell contacts (Supplementary Figure 2), suggesting it is the flagella that are required for the cell-cell contacts observed in the Δ*cj1387c* mutant. The Δ*pflA*-*cj1387c* double mutant possessed flagella but swarming motility was similar to the non-motile Δ*cj1387c-flaAB* strain (Figure 3). The Δ*pflA*-*cj1387c* double mutant exhibited “cell-trains” observed in the single Δ*cj1387c* mutant (Supplementary Figure 3). Taken together, this suggests that the “cell-train” phenotype, caused by inactivation of the *cj1387c* gene, is a result of interactions between flagella, but independent of a functional rotating flagellum.

### Cj1387c is a repressor of the downstream *cj1388* gene

**Figure 4:**
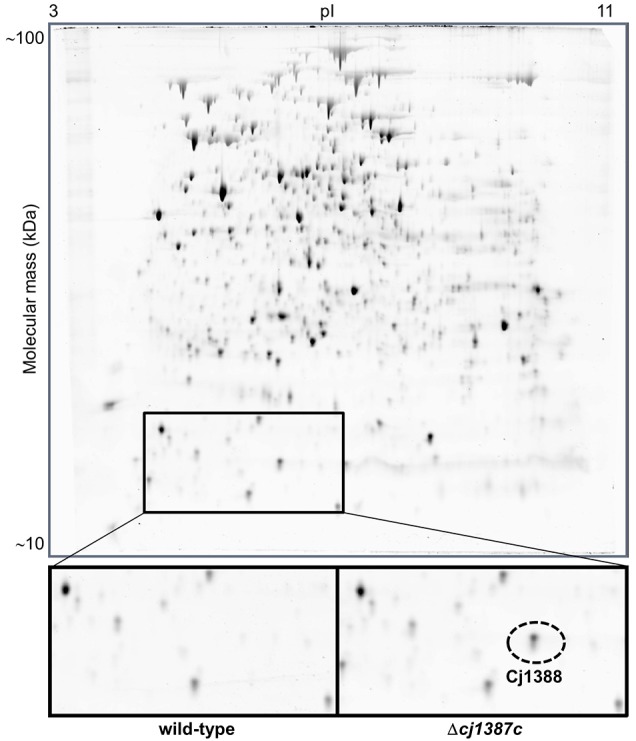
**Inactivation of** ***cj1387c*** **results in increased expressed of Cj1388**. The wild-type strain and the isogenic *cj1387c* mutant were grown for 12 h in Brucella medium, and total protein was separated by two-dimensional (2D) protein gel electrophoresis. A single protein difference was detected, which was identified as Cj1388. A representative 2D gel is shown, with the region containing Cj1388 magnified. The identity of Cj1388 was confirmed by excision of the protein fragment, trypsin digestion and mass spectroscopy. Cj1388 was identified using Mascot.

The Cj1387c protein contains a helix-turn-helix domain (Figure 1A), suggesting that this protein may bind DNA, and therefore have some regulatory function. Hence the Δ*cj1387c* mutant was compared to the wild-type strain using proteomics on two-dimensional gels. Only a single protein, identified as Cj1388, consistently showed differential expression when comparing the wild-type and Δ*cj1387c* mutant (Figure 4). This protein was observed in both the 6 and 12 h samples of the Δ*cj1387c* mutant, suggesting that Cj1387c represses *cj1388* transcription.

The Cj1388 protein comprises a single Endoribonuclease L-PSP (liver perchloric acid-soluble protein) domain (PF01042), see Figure 1, a domain that is predicted to inhibit protein synthesis via degradation of messenger RNA (Morishita et al., 1999). A search of the *C. jejuni* NCTC 11168 genome revealed the presence of a further Endoribonuclease L-PSP domain protein, Cj0327, which has 34% identity to Cj1388 (Figure 1B). The *cj0327* gene is not located adjacent to a putative regulatory protein, but is downstream of the SerC protein (phosphoserine aminotransferase) involved in serine biosynthesis (Figure 1A). A BLAST search of 75 *C. jejuni* and 45 *C. coli* genomes from the PATRIC database (either complete or WGS) revealed that Cj1387c, Cj1388 and Cj0327 were highly conserved in *C. jejuni* (74/75, 75/75, and 71/75 respectively), but that in *C. coli*, homologs of Cj0327 were completely absent, while the Cj1387c-1388 system is well conserved. A comprehensive search using the String database, using *cj1387c* as a query, showed that in organisms containing a *cj1387c* ortholog, while the genetic arrangement is varied, the *cj1388* ortholog is always either directly adjacent to the *cj1387c* ortholog, or separated by a single gene (Supplementary Figure 4).

### Cj1388 and Cj0327 are non-essential and are not required for motility

Insertional inactivation was used to determine the role of Cj1388 and Cj0327 proteins in *C. jejuni*. Mutants were created in the wild-type background. To make a double Δ*cj1388cj0327* mutant, the *cj0327* disruption construct (Cm^r^) was transformed into the Δ*cj1388* (Km^r^) background. Although *cj0327* has been proposed to be an essential gene (Stintzi et al., 2005), the *cj0327* mutants were viable and showed wild-type growth phenotypes. Ryu staining showed the presence of bi-polar flagella, as in the wild-type strain, and all mutants were motile (Figure 3). The Δ*cj1388* mutant had a significantly larger halo than the wild-type strain after both 24 and 48 h, whereas swarming motility of the Δ*cj0327* and Δ*cj1388cj0327* strains was not different from wild-type.

### Cj1387c, Cj1388, and Cj0327 contribute to autoagglutination

**Figure 5:**
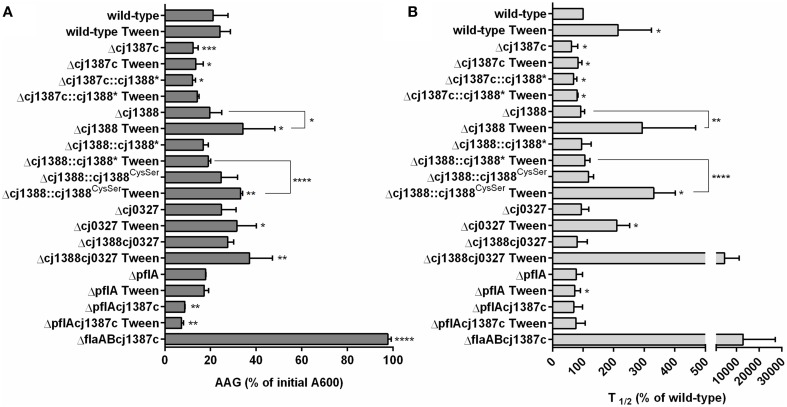
**Inactivation of** ***cj1387c*** **increases autoagglutination, while inactivation of** ***cj1388*** **and** ***cj0327*** **decreases autoagglutination in media supplemented with Tween-20**. AAG of cultures grown overnight in Brucella broth was measured over an 8 h time period and after 24 h at room temperature in air. The percentage of the initial A_600_ following 24 h **(A)** and t_1∕2_ of the steepest part of the AAG curve over the initial 8 h was calculated and expressed as a percentage of the wild-type **(B)**. Results show the mean of at least three biological replicates. Significantly different results, determined using either an unpaired *t*-test **(A)** or one-sample *t*-test **(B)** are indicated by asterisks (^*^p < 0.05, ^**^p < 0.01, ^***^p < 0.001, ^****^p < 0.0001).

Autoagglutination (AAG) was used to quantify flagella-mediated cell-cell contacts as observed in the Δ*cj1387c* strain, and assess the possible roles of Cj1388 and Cj0327 in this phenotype. *C. jejuni* is known to autoagglutinate, and this activity is known to be dependent on the flagella (Golden and Acheson, 2002). Overnight AAG (i.e., 24 h incubation, hereafter referred to as the final AAG measurement) was significantly lower in the Δ*cj1387c* strain compared to the wild-type: 10% of the starting A_600_ compared to 20% in the wild-type (Figure 5A). The AAG phenotype could be restored by expression of *cj1387c* from the constitutive *fdxA* promoter in pseudogene *cj0046* (Supplementary Figure 5). AAG was significantly reduced in a strain containing two copies of Cj1387c (WT::cj1387c^*^) compared to the wild-type. This may be explained by the low level of *cj1388* transcript that was detected by RNA-seq analysis (Porcelli et al., 2013; Handley et al., 2015), with a RPKM value of 1.97. We hypothesize that overexpression of *cj1387c* from two gene copies might therefore result in full repression of *cj1388* transcript, resulting in decreased AAG compared to the wild-type. The T_1∕2_, describing the steepest part of an 8 h AAG assay, was 60% of the wild-type in the Δ*cj1387c* strain (Figure 5B). The increased AAG phenotype of the Δ*cj1387c* strain is consistent with the microscopy observations showing flagella-mediated cell-cell contacts.

To assess the strength and nature of the flagella-flagella interactions, AAG assays were repeated in media supplemented with sub-inhibitory concentrations of various detergents (Svensson et al., 2009). Assays in media supplemented with either 0.00025% SDS or 0.05% Triton X-100 had no effect on either AAG rate or final AAG levels (data not shown). However, AAG in media supplemented with 0.002% Tween-20 did affect AAG in a strain-dependent manner (Figure 5). Tween-20 marginally slowed the AAG rate of the wild-type, and this effect could be increased by increasing the level of Tween-20 (data not shown). The Δ*cj1387c* strain showed no response to Tween-20, either in rate or final AAG. In Tween 20-supplemented media, the rate of AAG of the Δ*cj1388* strain was significantly slower than the wild-type (almost 300% of that observed in the wild-type) and final AAG was around 34% of the initial A_600_. Complementation of the *cj1388* mutant with constitutively expressed *cj1388* restored the wild-type phenotype. The final AAG measurements of the Δ*cj0327* and Δ*cj1388-cj0327* strains were significantly different from the wild-type in Tween 20-supplemented media. Combining both the *cj1388* and *cj0327* mutations had a dramatic effect on AAG rate: the Δ*cj1388-cj0327* double mutant behaved essentially as a Δ*cj1387c-flaAB* double mutant over the course of an 8 h AAG assay. AAG in the Δ*cj1387c-flaAB* strain is totally abolished due to absence of flagella. AAG of the Δ*pflA* strain was similar to the wild-type, suggesting that flagella rotation is not required for AAG. Like in the Δ*cj1387c* strain, AAG in the Δ*pflA-cj1387c* double mutant was increased, relative to the wild-type. The Δ*pflA-cj1387c* double mutant actually showed a significantly lower final AAG value compared to the single Δ*cj1387c* strain.

### Cj1388 forms a disulfide linked dimer

**Figure 6:**
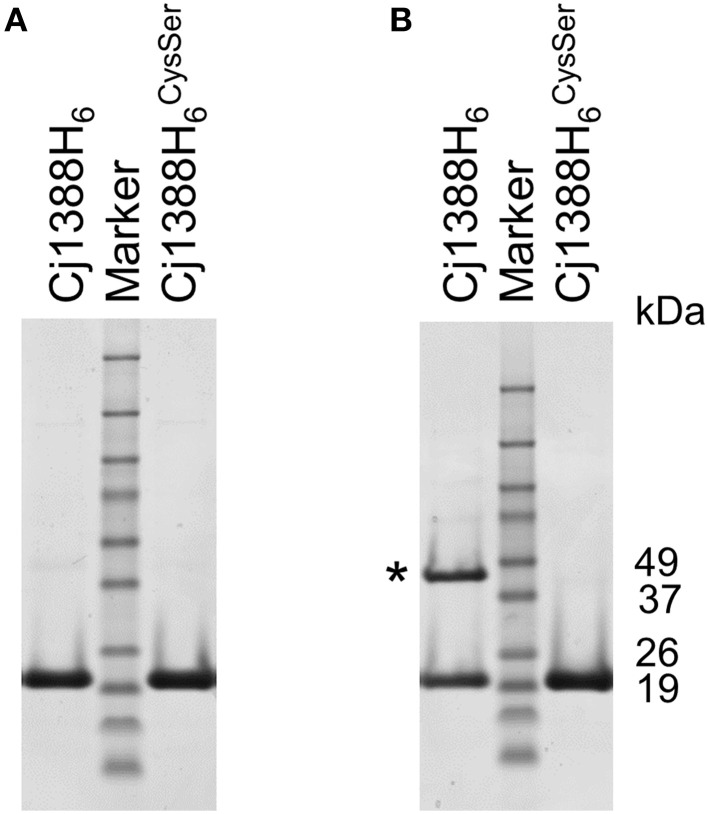
**Cj1388 forms a dimer via a disulfide bridge. Cj1388H_6_ or Cj1388 H^CysSer^_6_ were cloned into pET28a and induced with IPTG**. SDS-PAGE in non-reducing conditions **(B)** shows the appearance of a protein fragment twice the size of the purified protein (marked with an asterisk) not visible in reducing conditions **(A)** or in samples containing Cj1388H^CysSer^_6_.

Examination of the Cj1388 primary sequence revealed a single cysteine residue at position 71 (Figure 1B). Reasoning that this residue may be involved in disulfide bridge formation, two over-expression constructs were made. Firstly, *cj1388* was cloned into pET28a to make an N-terminal translationally-fused his-tagged protein. The cysteine codon was then substituted for a serine codon (TGC to AGC) using site-directed mutagenesis. Both constructs were transformed into *E. coli* strain BL21 (DE3) and protein expression was induced with IPTG. SDS-PAGE analysis showed that both expression strains resulted in the clear over-expression of a soluble protein of the expected size. Repeating SDS-PAGE analysis of the Cj1388H_6_ fractions in non-reducing conditions showed the appearance of a 44 kDa protein band, calculated to be 2.4 times the size of the purified protein observed in reducing conditions (Figure 6). The identity of both the higher and lower molecular protein bands was confirmed to be Cj1388 using LC-MS-MS ion search by Mascot with full coverage of the predicted protein sequence when using a chymotrypsin digest for the reduced and alkylated protein. Searching the upper band digest for the presence of the disulfide-linked peptide using the Mass Matrix program disappointingly failed to identify the crosslinked peptide species. SDS-PAGE analysis of the Cj1388^Cys71Ser^ fractions in non-reducing conditions did not show the 44 kDa fragment. Thus, Cj1388 can form a dimer where subunits are connected via a disulfide bridge. To test if dimer formation is required for function, the *cj1388* complementation construct was subjected to site-directed mutagenesis to substitute Cys71 for serine, as in the expression construct. This construct was then transformed into the Δ*cj1388* strain to make strain *cj1388*::*cj1388*^*Cys*71*Ser*^. While the *cj1388* complement strain can rescue the Tween20 phenotype of the Δ*cj1388* mutant, the *cj1388*::*cj1388*^*Cys*71*Ser*^ cannot, suggesting that dimer formation may be required for function of Cj1388 in modulating flagella interactions in media containing Tween 20 (Figure 5).

Given that Cj1388 is predicted to consist of a single endoribonuclease domain, its ability to degrade nucleic acid was tested. Purified Cj1388 did not show any nuclease activity on *C. jejuni* ribosomal RNA or DNA and lysates from IPTG-induced cultures expressing Cj1388 were negative on DNase indicator agar plates (Gaasbeek et al., 2010), data not shown.

### Cj1388 and Cj0327 have a role in virulence in the *Galleria mellonella* model

**Figure 7:**
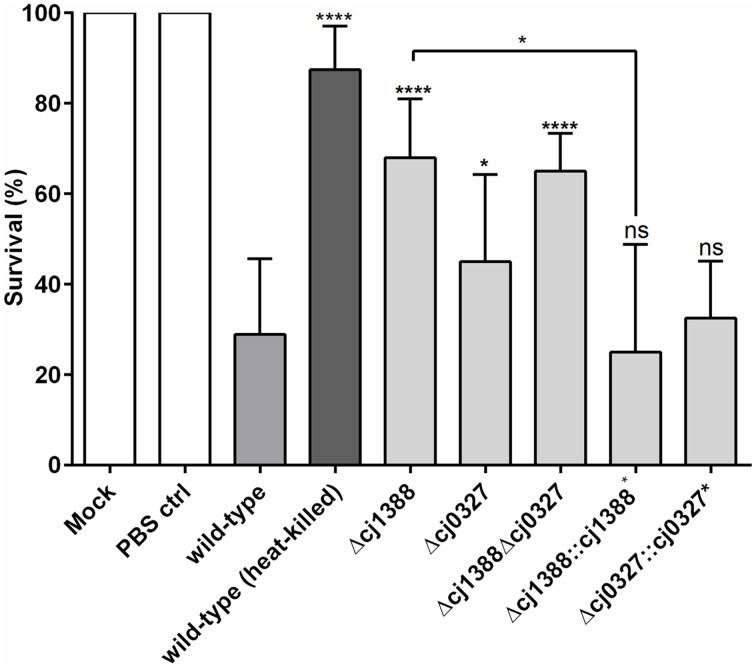
**Strains lacking Cj1387c, Cj1388, and/or Cj0327 are attenuated in the** ***Galleria*** **wax moth larvae model**. Galleria larvae were injected with 10 μl of cells (1 × 10^6^ cells) harvested from Skirrow plates and washed in PBS. Mock injection, injection with PBS, and injection with heat-killed *C. jejuni* were included as negative controls. Results show the mean of at least four biological replicates, each containing 10 larvae. Significantly different results, determined using unpaired *t*-tests, are shown using asterisks (^*^p < 0.05, ^****^p < 0.0001).

Bacterial flagella are known to be highly antigenic (Hayashi et al., 2001; Smith et al., 2003), and motility in *C. jejuni* is a known virulence factor (Lee et al., 1986; Szymanski et al., 1995; Carrillo et al., 2004). Given that the Cj1387c-1388 system affects AAG, which is dependent on functioning flagella, we sought to assess the role of Cj1388 and Cj0327 on virulence using the *Galleria mellonella* virulence model (Champion et al., 2010). The Δ*cj1388*, Δ*cj0327*, and Δ*cj1388*-*cj0327* strains showed attenuation in *Galleria* larvae, with the Δ*cj1388* strain showing the greatest level of attenuation (Figure 7). The virulence phenotype of the Δ*cj1388* and Δ*cj0327* strains could be restored by complementation with their cognate gene.

## Discussion

The regulatory cascade controlling expression of flagellar genes in *Campylobacter* is increasingly well understood; however, there are fewer insights into the regulatory control of post-translational modification of flagella. The paradigm of sensing the environment followed by modulating behavior, enzyme activity, or gene expression to adapt to those changes is well established. Thus, it is not surprising that post-translational control of flagella should also be subject to environmental control. In this study we have identified a new PAS-domain-containing repressor protein, which affects flagella-flagella interactions resulting in higher levels of autoagglutination. Proteomics analysis suggests that this regulatory protein represses expression of the adjacent downstream gene encoding a dimeric effector protein Cj1388. This protein, in concert with a homolog encoded in a different region of the chromosome, affects autoagglutination and may play a role in virulence. Homologs of Cj1387c and Cj1388 in other organisms are always found linked suggesting that this system always functions in concert.

PAS domains are known to sense diverse environmental signals and participate in dimerization (Ma et al., 2008; Slavny et al., 2010). Unlike the PAS domain found in other *Campylobacter* proteins, the (YheO-like) PAS domain found in Cj1387c is only found in bacteria and exhibits a very limited architectural diversity. Very little is known about this member of the PAS family; the sensed stimuli or signal, if any, for Cj1387c is not known. The Cj1388 effector protein appears to be a dimer, formed via a disulfide bridge between two monomers. This suggests that there could be a link to redox control; indeed, redox sensing in PAS domain sensors is well established (Hill et al., 1996; Xie et al., 2010; Sousa et al., 2013).

Deleting Δ*cj1387c* resulted in a clear “cell-train” morphology. Ryu flagella staining suggested that the cells were connected via flagella and accordingly, combining the *cj1387c* disruption with a *flaAB* mutant abolished cell chain formation. Flagella rotation, however, is dispensable for cell chain formation as the *cj1387c* mutant in a paralyzed flagella background showed the “cell-train” morphology and rapid AAG. The paralyzed flagella mutant in strain 81–176 is known to show normal AAG (Guerry et al., 2006) and we confirm this observation for the NCTC 11168 background. Interaction between flagella during AAG is therefore most likely to occur via a physical interaction between flagella filaments. Interestingly, cell chains are still motile, and chains of up to three cells could be observed with rapid darting motility. Given that flagella rotation is governed by CheY and subject to chemotactic control, this raises the intriguing question as to which cells in a cell-chain determine direction and how does directional dominance arise? The role of these cell chains in the normal physiology and survival of *C. jejuni* is not known. The flagella-flagella interactions may merely be a consequence of disrupting the normal flagella glycan decoration pathways, which are known to be essential for flagella assembly and function (Goon et al., 2003; Asakura et al., 2013). Clearly, disrupting *cj1387c*-*88* does not abolish glycosylation, as the cells still possess flagella and are motile and no gross differences in glycoprotein could be detected by Alcian Blue staining. Changes in glycosylation decoration of the flagella are however the most likely explanation for the changes in AAG observed in this study. AAG is known to be important for microcolony formation at the initiation of biofilm formation (Cole et al., 2004; Boddey et al., 2006; Moreira et al., 2006), and in *C. jejuni*, flagella are required for robust biofilm formation (Joshua et al., 2006; Reuter et al., 2010). The biofilm phenotype of the Δ*cj1387c* and Δ*cj1388* strains was not different from the wild-type as assessed by microscopy and crystal violet staining.

Both Cj1388 and Cj0327 are small (13 kDa) proteins consisting of single endoribonuclease L-PSP domain. This domain was first characterized as liver perchloric acid-soluble protein (hence L-PSP) from Rat liver and shown to inhibit protein synthesis (cell free system using rabbit reticulocyte lysate) and degrade *in vitro* transcribed mRNA (Oka et al., 1995; Morishita et al., 1999). Non-reducing SDS-PAGE and substitution of the single cysteine residue in Cj1388 demonstrated that this protein likely forms a disulfide stabilized dimer. A complement construct carrying the Cys71Ser substitution also failed to complement the AAG-Tween phenotype, suggesting that dimerization is required for function. If indeed these proteins do function to degrade mRNA, this suggests that post-transcriptional regulation may play a role in regulating flagella function. While both Cj1388 and Cj0327 score as significant for a Ribonuclease L-PSP domain, they are only 36% identical. Cj0327 has two cysteine residues, and it is unknown if these contribute to inter- or intra-domain disulfide bridge formation. Based on the levels of AAG in Tween-supplemented media, disrupting either gene has the same consequence (reduced AAG) and this phenotype is most extreme when both mutations are combined. Degenerate function has been seen previously in *C. jejuni*: the Cet energy taxis system can function with either CetB and CetC (Reuter and van Vliet, 2013) and both FlaA and FlaB flagella proteins are incorporated into the flagella filament (Logan et al., 1987; Guerry et al., 1991). Moreover, *C. jejuni* strain RM1221 contains three extra-cellular DNases (Gaasbeek et al., 2010). The presence of two Ribonuclease L-PSP proteins in *C. jejuni* is an example of functional redundancy, although *C. coli* has only the single *cj1387c*-linked gene. Also, based on the String analysis, Cj0327 homologs are much less common than the linked *cj1387c*-*88* system.

Disrupting both *cj1388* and *cj0327* genes resulted in significant attenuation in the *Galleria* wax moth larvae model, and virulence in this model was restored by constitutive *in trans* expression of Cj1388. It is well established that changes in glycosylation and AAG affect adhesion to and invasion of human intestinal cells (Guerry et al., 2006). Although flagella from *Campylobacter* and *Helicobacter* lack TLR5-recognition sites (Galkin et al., 2008), the sheer size of the flagella suggests that it will be highly antigenic. As part of the insect immune system, N-acetylglucosamine-specific lectins are known to facilitate phagocytosis by the haemocyte cells (insect phagocytes) (Kavanagh and Reeves, 2004) and this process is further augmented by the action of lysozyme, which degrades bacterial peptidoglycan exposing lectin-specific molecules such as teichoic acid and lipomannans (Wilson and Ratcliffe, 2000). Therefore, changes in surface polysaccharides, on either the flagella or cell might be expected to influence virulence in an insect model. The Cj1388-Cj0327 proteins may therefore represent hitherto unknown virulence determinants.

**Figure 8:**
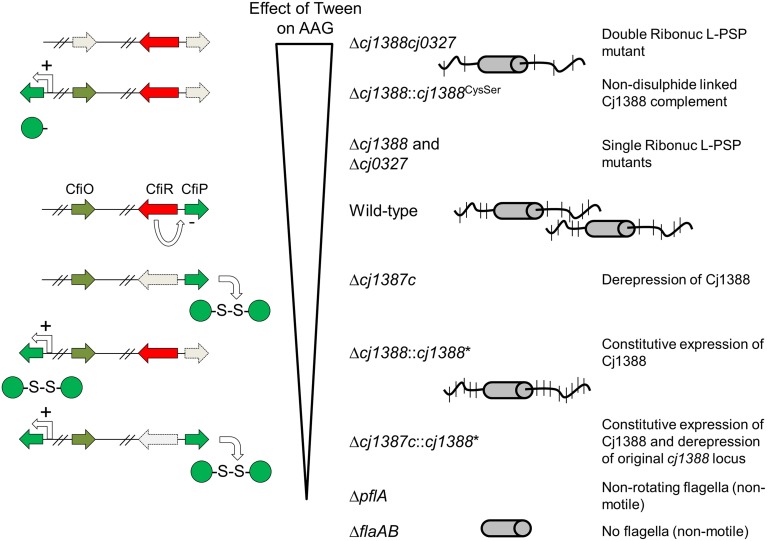
**Model showing the effect of disrupting the** ***cj1387c*****-*****cj1388*****-*****cj0327*** **genes on autoagglutination (AAG)**. Strains are shown ranked in the order in which AAG is decreased in media supplemented with Tween 20 compared to un-supplemented media. Cj1388 expression is proposed to be de-repressed when *cj1387c* is inactivated resulting in greater AAG, which cannot be disrupted by Tween. Deleting both *cj1388* and/or *cj0327* results in a Tween-dependent decrease in AAG. Complementation by Cj1388 rescues this phenotype, and this activity is dependent on a single cysteine residue in Cj1388, which forms a dis- sulfide-linked dimer *in vitro*. AAG is dependent on flagella glycan decoration, suggesting that Cj1388 and Cj0327 have a role in mediating glycan modification of flagella.

In summary, we have identified a novel system that affects flagella-flagella interactions. We propose to designate the Cj1387c repressor as CfiR (Campylobacter flagella Interaction Regulator) and name the effectors as CfiP (Cj1388) and CfiQ (Cj0327). Proteomics analysis shows that CfiP is repressed by CfiR. We present a model whereby CfiPQ control flagella glycan decoration, which influences cell aggregation and virulence (Figure 8). Deletion of CfiPQ results in attenuation in the Galleria model and a disruption of flagella-mediated autoagglutination by the surfactant Tween-20; derepressed expression of disulfide-linked CfiP dimers results in tightly connected “cell-trains” mediated by the flagella, and increased aggregation. CfiRP homologs are found in other bacterial species, and always linked on the chromosome. Therefore, this system, acting in concert, may control glycan modification in other bacteria, which may influence virulence and immuno-modulation in pathogens such as *S. pyogenes, S. enterica, V. parahaemolyticus*, and *Y. pestis*.

### Conflict of interest statement

The authors declare that the research was conducted in the absence of any commercial or financial relationships that could be construed as a potential conflict of interest.
