# Supplementary material for: A PAS domain-containing regulator controls flagella-flagella interactions in Campylobacter jejuni
Source: Front Microbiol. 2015 Jul 30;6:770. doi: 10.3389/fmicb.2015.00770 (PMC4519771; doi:10.3389/fmicb.2015.00770)
Supplement: Supplementary file 1 [file Table1.DOCX]

***Supplementary Material***

**A PAS Domain-Containing Regulator Controls Flagella-Flagella Interactions in *Campylobacter jejuni***

**Mark Reuter^1,*^, Paula M. Periago^2, 3^, Francis Mulholland^1^, Helen L. Brown^1,4^, and Arnoud H.M. van Vliet^1^**

^1^Institute of Food Research, Gut Health and Food Safety programme, Norwich Research Park, Norwich NR4 7UA, United Kingdom.

^2,3^Dpto. Ingeniería de Alimentos y del Equipamiento Agrícola, Campus de Excelencia Internacional Regional ‘‘Campus Mare Nostrum’’, Escuela Técnica Superior de Ingeniería Agronómica, Universidad Politécnica de Cartagena, Spain; Instituto de Biotecnología Vegetal, Campus de Excelencia Internacional Regional ‘‘Campus Mare Nostrum’’, Universidad Politécnica de Cartagena, Spain.

^4^Cardiff School of Health Sciences, Cardiff Metropolitan University, Llandaff Campus, Cardiff, CF5 2YB, UK

* **Correspondence:**

Institute of Food Research

Norwich Research Park

Colney Lane

Norwich NR4 7UA

United Kingdom.

Email: mark.reuter@ifr.ac.uk

## Supplementary Tables

**Supplementary Table 1 | Primers used in this study**

| **Name** | **Sequence ^a^** | **Description** |
| --- | --- | --- |
| cj1388_flankFwd | 5'-CCTAAAATggtACCCATATCTCC-3’ | Upstream primer to amplify *cj1388 locus* plus ≈500bp flanking sequence. |
| cj1388_flankRev | 5'-CCATGActgCAgTTCCACTTCC-3' | Downstream primer to amplify *cj1388* locus plus ≈500bp flanking sequence. |
| cj0327flankFwd | 5'-CCTAAAACTAgagCTCCTTTGATTGTGG -3' | Upstream primer to amplify *cj0327* plus ≈500bp flanking sequence. |
| cj0327flankRev | 5'- GGAGCTGAAAAAgCAtGcTCAATTATGG -3' | Downstream primer to amplify *cj0327* plus ≈500bp flanking sequence. |
| cj1388_inverseStart | 5'-ctagggatccCCATTTACTTCTCTGTAAGC -3' | N-terminal inverse PCR primer for replacing *cj1388* with an antibiotic cassette. |
| cj1388_inverseEnd | 5'-ctagggatccCCTTATCCAGCAAGAAGTGC-3' | C-terminal inverse PCR primer for replacing *cj1388* with an antibiotic cassette. |
| cj0327InverseIBamHI | 5'-ctagggatccGGTTCAAAAGCCACTTGTCCTGC -3' | N-terminal inverse PCR primer for replacing *cj0327* with an antibiotic cassette. |
| cj0327InverseIIBamHI | 5'-ctagggatccGCTTTAGCAGAGGGTTATTTGG-3' | C-terminal inverse PCR primer for replacing *cj0327* with an antibiotic cassette. |
| cj0326Fwd | 5'-GGAAAGTAATTTCGATCATATTCC-3' | Primer anneals outside *cj0327* flanking region and used to confirm insertional inactivation of *cj0327*. |
| cj0328Fwd | 5'-GCTAAAGCCTTAGTAGAAAGTGG-3' | Primer anneals outside *cj0327* flanking region and used to confirm insertional inactivation of *cj0327*. |
| cj1388comp_FwdBspHI | 5'-GGAGAATTCATGagcAACTATCCAAAG-3' | Primer used to amplify *cj1388* for complementation in pCfdxA. The 2^nd^ codon is altered from TCA to AGC; both encode Serine. |
| cj1388comp_RevBspHI | 5'- GAAAAAtCAtgACCTAGCATGTTTTATCC-3' | Reverse primer used to amplify *cj1388* for complementation in pCfdxA. |
| Cj1387cCompFwd | 5'-GAAGGCAGTTccATGGATGAGGG -3 | Primer used to amplify *cj1387c* for complementation in pKfdxA. |
| Cj1387cCompRev | 5'-CCTTATACcaTgGCTATTATGTTTGG-3’ | Reverse primer used to amplify *cj1387c* for complementation in pKfdxA. |
| Cj0327Comp_Fwd | 5'-CAAGGAAAATATtCATGATAAAGCG-3' | Primer used to amplify *cj0327* for complementation in pKfdxA. |
| Cj0327Comp_Rev | 5'-GGATCTGCGCTcaTgAAATTTGG-3' | Reverse primer used to amplify *cj0327* for complementation in pKfdxA. |
| pflAFwdScreen | 5'-GCTTATAGGTGCTTTTGCGGCTC-3’ | Primer anneals in *cj1566c* and was used to confirm *pflA* insertional inactivation. |
| pflAKOR | 5'-CAATCTTTAGAAGAAACTGCAGCAGCT-3’ | Primer anneals upstream of *cj1565c* (*pflA*) and was used to confirm *pflA* insertional inactivation. |
| KmReadOut | 5'-CGGGGAAGAACAGTATGTCGAGC-3’ | Reads out from the 3’ end of the Kanamycin resistance cassette. |
| KmPrReadOut | 5’-GCGATATCTTCTATATAAGCGTACCG-3’ | Reads out from the 5’ end of the Kanamycin resistance cassette. |
| CatReadOut | 5’- CGTTTGTGACGGCTTTCATGTTTGC-3’ | Reads out from the 3’ end of the Chloramphenicol resistance cassette. |
| CatPrReadOut | 5’-GGTCGAAATACTCTTTTCGTGTCC-3’ | Reads out from the 5’ end of the Chloramphenicol resistance cassette. |
| 0046Fcheck3 | 5’-GCAGAGCACTTGATTTTAGTGTGTGC-3’ | Primer for checking inserts into pseudogene *cj0046* used for complementation (upstream primer). |
| 0046Rcheck | 5’-CCTGGAGAAGTATTAGATAGTAGCGG-3’ | Primer for checking inserts into pseudogene *cj0046* used for complementation (downstream primer). |
| cj1388pET_Fwd | 5'-GGAGAAcatATGTCAAACTATCCAAAG-3' | N-terminal primer for cloning *cj1388* into pET28a |
| cj1388pET_Rev | 5'-GAAAAAAggATcCCTAGCATGTTTTATCC-3' | C-terminal primer for cloning *cj1388* into pET28a |
| 1388Cys-SerDpnI1 | 5’-CCACTaGCTTTTTAGCTGATATTAATG-3’ | cys71ser site-directed mutagenesis primer (cj1388) |
| 1388Cys-SerDpnI2 | 5’-GCTAAAAAGCtAGTGGTTTTAATCAC-3’ | cys71ser site-directed mutagenesis primer (cj1388) |

^a^ Restriction enzyme sites that are introduced for cloning purposes are underlined. Mismatched bases are in lowercase.

## Supplementary Videos

Supplementary Video 1 | Cell chains formed by the *cj1387c* strain are motile. The *cj1387c* strain was grown overnight in Brucella broth under microaerobic conditions at 37°C and mounted directly on a twin-frost microscope slide. Movies were recorded, and short clips were made using ImageJ. iMovie was used to compile the video clips.

- 1. **Supplementary Figures**

**Supplementary Figure 1** | **Comparison of domain architecture and total number of sequences for the PAS (Per-ARNT-Sim) protein domain family**. Cj1387c contains a PAS6 (YheO-like) domain (PF08348, orange box), which exhibits limited architectural variation in comparison with the other members of the PAS family. All other PAS domains found in *C. jejuni* are classified as PAS9 domains (blue box). For the PAS family, the number of sequences is observed to correlate with architecture variation (coefficient of determination (R^2^) = 0.91).

**Supplementary Figure 2** | **Flagella are required for cell chain formation resulting from the Δ*cj1387c* disruption**. The *flaABcj1387c* strain was grown overnight in Brucella broth under microaerobic conditions at 37°C and mounted directly on a twin-frost microscope slide. Fresh Ryu stain was applied to the coverslip and cells photographed at x100 magnification.

**Supplementary Figure 3 |** **Cell chain formation is independent of functional rotating flagella**. The Δ*pflAcj1387c* strain was grown overnight in Brucella broth under microaerobic conditions at 37°C and mounted directly on a twin-frost microscope slide. Fresh Ryu stain was applied to the coverslip and cells photographed at x100 magnification.

**Supplementary Figure 4 |** **Orthologs of Cj1387c and Cj1388 show genetic linkage**. A. The String database was used to show homologs of Cj1387c. In all cases, organisms containing a homolog to Cj1387c also contained an adjacent Cj1388 homolog, although the configurations varied (Sets 1 – 7). This was also the case in examples of gene duplication (Set 7). In some instances, the Cj1387c and Cj1388 homologs were separated by a single gene, with the exception of *Photobacterium profundum* and *Geobacillus* sp. Y412MC10 (two intervening proteins), and *Atopobium parvulum* DSM 20469 (nine intervening proteins). The intervening proteins vary in their predicted function, and represent different cellular functions (25 different orthologous group clusters (COGs)). Homologs to the Cj0327 protein are less common; the String database shows only 23 homologs in bacteria and one Archaeal homolog (in *Natronomonas pharaonis*). Homologs of Cj0327 are most common in *C. jejuni*, *Y. pestis*, and *Y. pseudotuberculosis* (12/24 instances).

B. A pie chart showing the distribution of the different genetic configurations reveals that Set 2 is the most common genetic arrangement (a Cj1387c homolog followed by a Cj1388 homolog).

Supplementary Figure 5 | Complementation of the Δ*cj1387c* mutant by constitutively expressing Cj1387c rescues the AAG phenotype. A construct expressing Cj1387c from the constitutive *fdxA* promoter was inserted into pseudogene *cj0046c* in both wild-type and Δ*cj1387c* strains. A strain containing two copies of Cj1387c (WT::cj1387c*) shows significantly less AAG compared to wild-type. Complementing the *cj1387c* inactivation by constitutively expressing Cj1387c (Δ*cj1387c*::*cj1387c**) rescues the AAG phenotype: AAG is not statistically different from the wild-type but is statistically different from the Δ*cj1387c* strain. Significantly different results were determined using an unpaired t-test (* = p < 0.05, ** = p < 0.01).

**
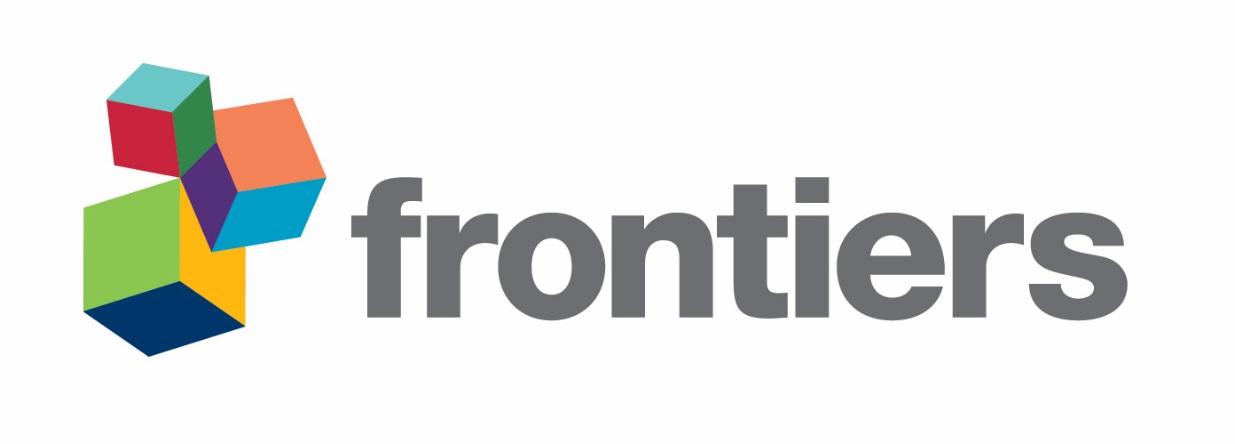
**
