# Supplementary figures and images for: A PAS domain-containing regulator controls flagella-flagella interactions in Campylobacter jejuni
Source: Front Microbiol. 2015 Jul 30;6:770. doi: 10.3389/fmicb.2015.00770 (PMC4519771; doi:10.3389/fmicb.2015.00770)

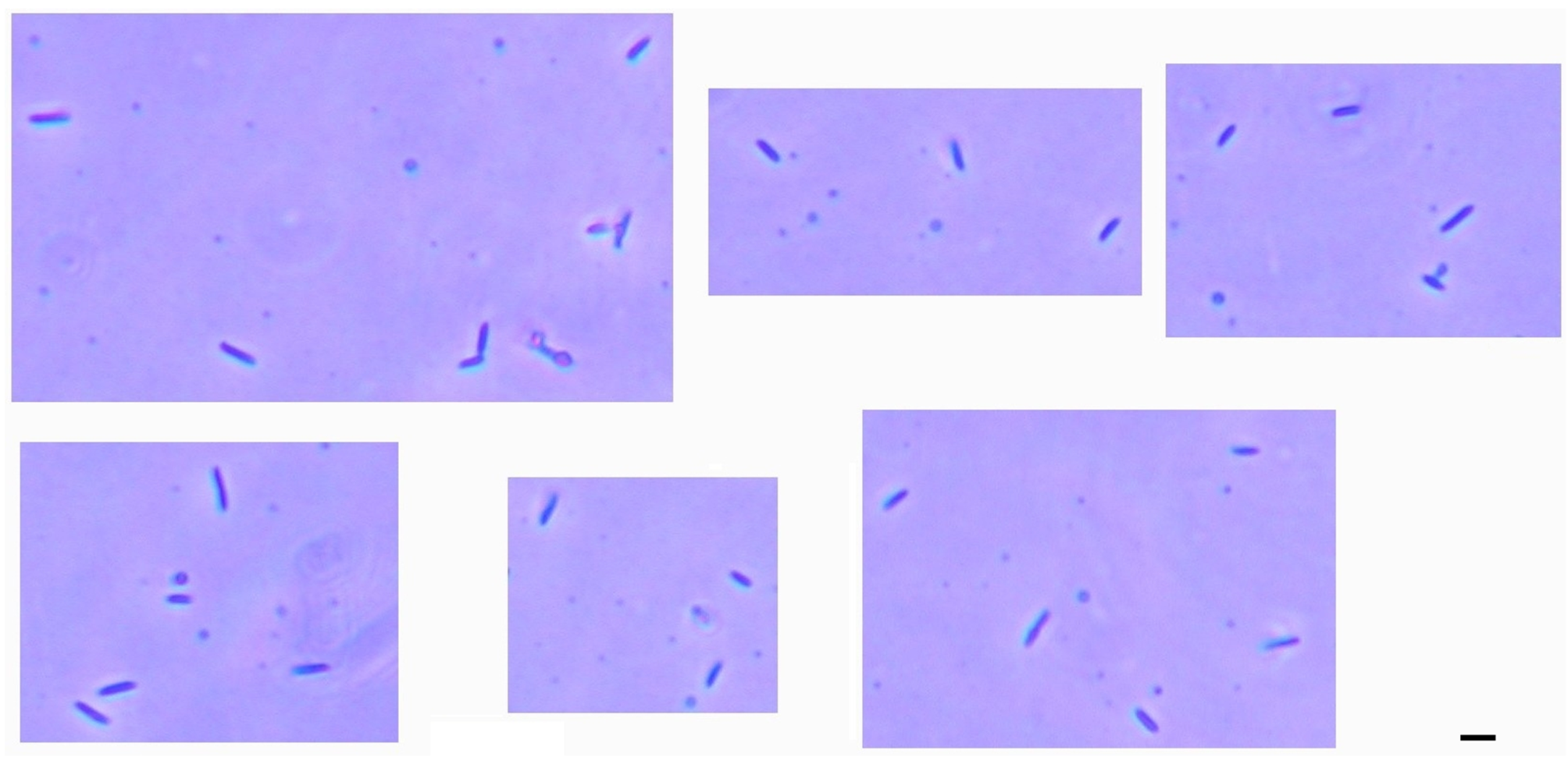

Supplement: Supplementary file 4 [file Image2.TIF]

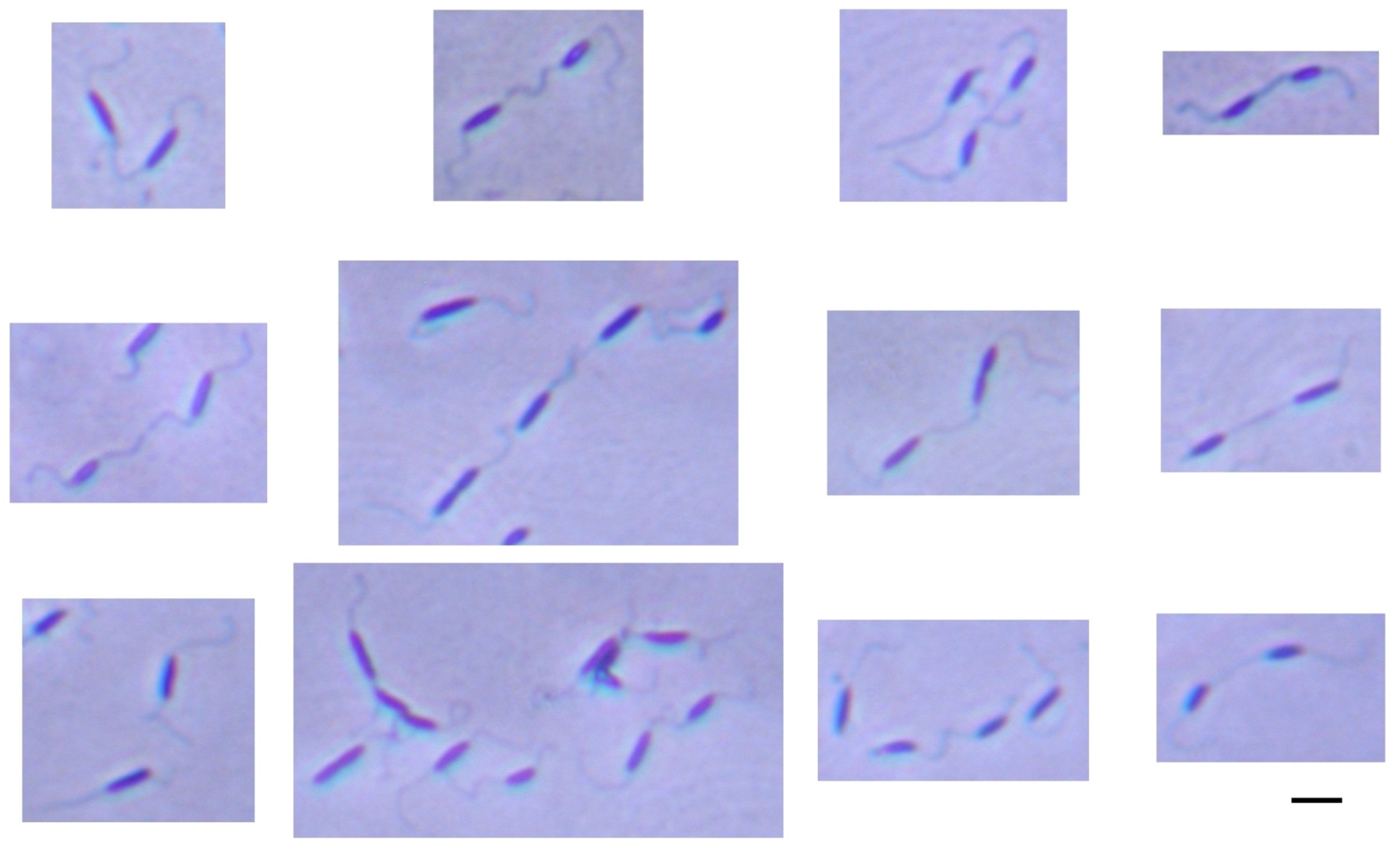

Supplement: Supplementary file 5 [file Image3.TIF]

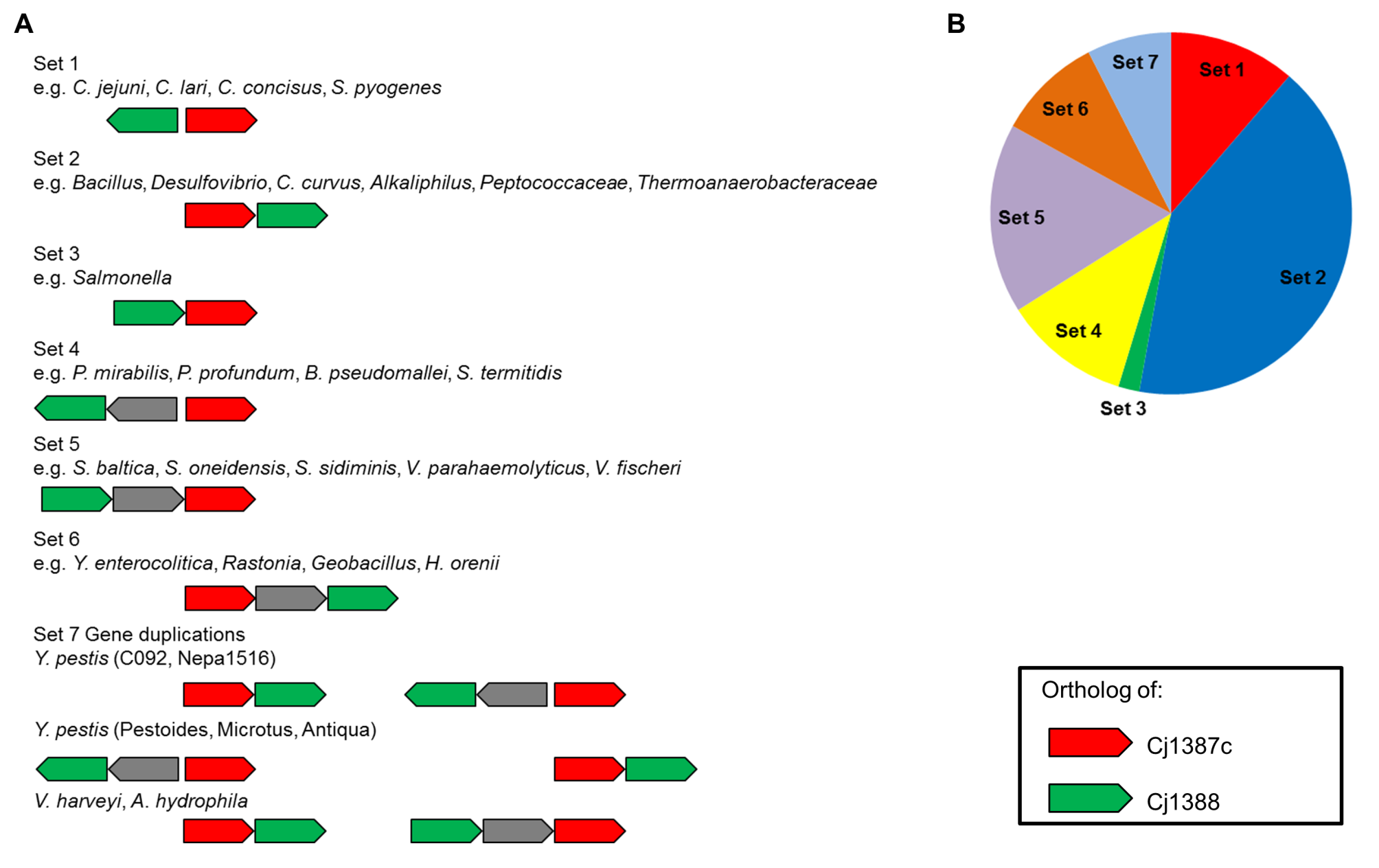

Supplement: Supplementary file 6 [file Image4.TIF]

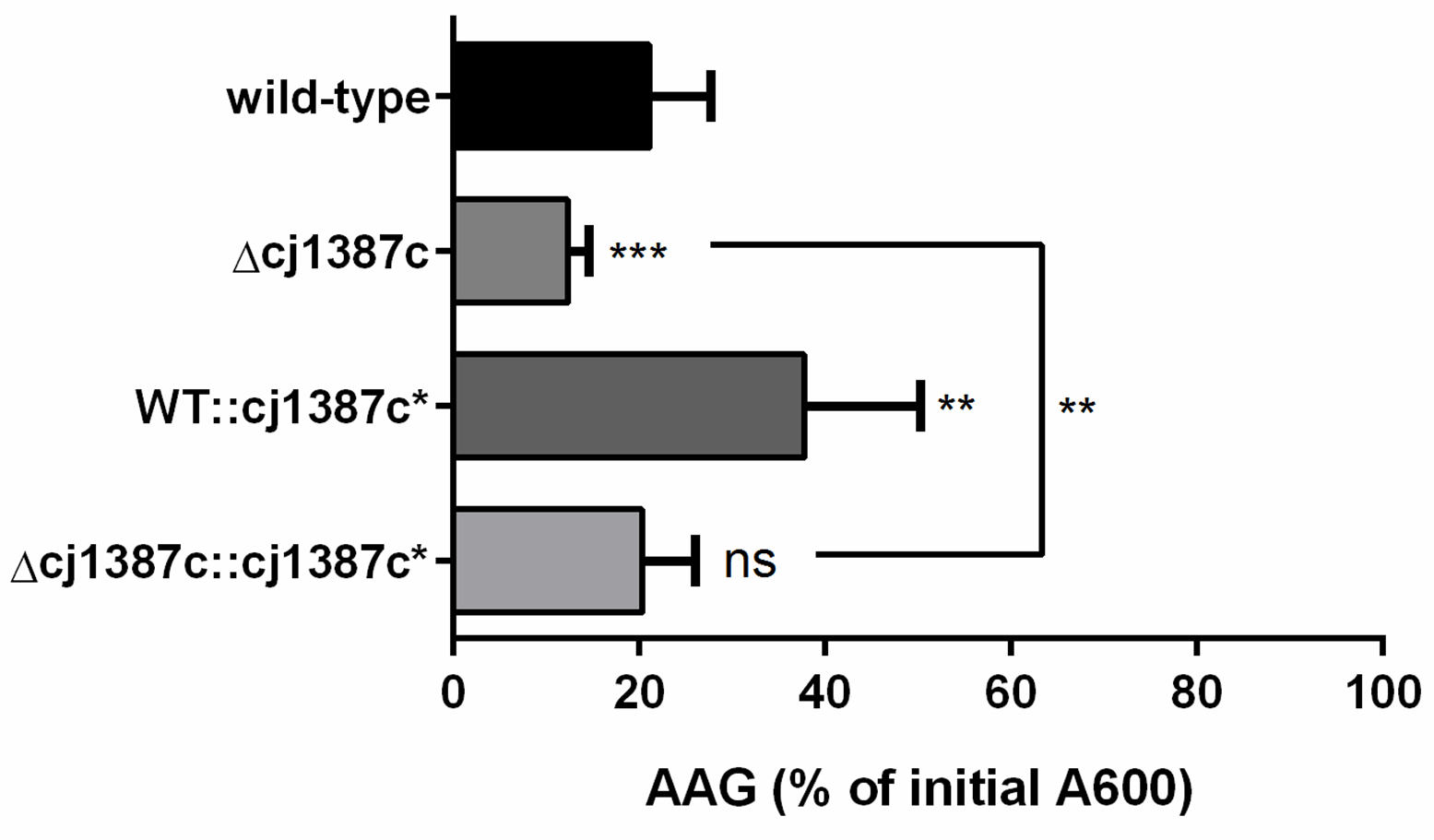

Supplement: Supplementary file 7 [file Image5.TIF]
